# Photodynamic treatment in glioma: Metabolic and structural evaluation after therapy

**DOI:** 10.1111/php.70032

**Published:** 2025-09-07

**Authors:** Marina Gabriela Teixeira, Luciana Maria Cortez Marcolino, Juliana Guerra Pinto, Rainara Moreno Sanches de Almeida, Isabelle Ferreira, Juliana Ferreira‐Strixino

**Affiliations:** ^1^ Photobiology Applied to Health (PhotoBioS Lab) University of Vale do Paraíba São Paulo Brazil

**Keywords:** chlorine, cytometry, gliosarcoma, photodynamic therapy

## Abstract

Gliomas are malignant tumors of the central nervous system, and one severe variant is called gliosarcoma. Photodynamic therapy (PDT) is a technique that stands out in the oncology area for minimizing side effects for the patient, triggering cell death at the site of irradiation, and can be used concomitantly with conventional treatments. This study aimed to evaluate the interaction of chlorine e6 with the cytoskeleton and mitochondria, as well as morphological changes and the death mechanism triggered after PDT. Chlorin e6 was used at concentrations of 200, 12.5, and 6.25 μg/mL, and cytoskeletal changes were analyzed by alpha‐tubulin staining and mitochondrial membrane potential (MMP) analysis by JC‐1 and Rhodamine 123 in flow cytometry. Surface features were examined using scanning electron microscopy, and the type of cell death mechanism was determined by flow cytometry with annexin and propidium iodide. Changes in the cytoskeleton were observed after PDT. Cytometry showed that cell death occurred predominantly via the apoptosis pathway, followed by the necrosis pathway. Chlorin e6 associated with PDT causes damage to gliosarcoma cells, regardless of concentration, showing cytoskeletal disruption, a decrease in MMP, and the percentage of cell death varies according to the concentration of PS.

## Graphical abstract

PDT with chlorin e6 reduced mitochondrial membrane potential and induced gliosarcoma cell death, mainly via apoptosis and subsequently necrosis. Microscopy and flow cytometry confirmed cytoskeletal disorganization and morphological changes. These findings highlight PDT with Ce6 as a promising therapeutic alternative for gliosarcomas, showing strong cytotoxic and mitochondria‐targeted effects.

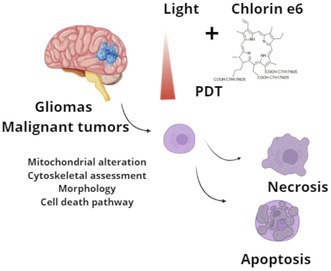

**Abbreviations**
APCs: antigen‐presenting cells
ATP: adenosine triphosphate
Bax: Bcl‐2‐associated X protein
Bcl‐2: B‐cell lymphoma 2
BD: Becton Dickinson
Ce6: chlorin e6
CLM: Central de Laboratórios Multiusuários
CNPq: National Council for Scientific and Technological Development
CNS: central nervous system
DAMPs: damage‐associated molecular patterns
DAPI: 4′,6‐diamidino‐2‐phenylindole
DMEM: Dulbecco's Modified Eagle Medium
FAPESP: São Paulo Research Foundation
FBS: fetal bovine serum
FITC: fluorescein isothiocyanate
GPX4: glutathione peroxidase 4
GS: gliosarcoma
GSH: glutathione
H₂O₂: hydrogen peroxide
HMGB1: high mobility group box 1
ICD: immunogenic cell death
JC‐1: 5,5′,6,6′‐tetrachloro‐1,1′,3,3′‐tetraethylbenzimidazolylcarbocyanine iodide
LED: light emitting diode
MLKL: mixed lineage kinase domain‐like
MMP: mitochondrial membrane potential
PARP‐1: poly (ADP‐ribose) polymerase 1
PBS: phosphate‐buffered saline
PDT: photodynamic therapy
PI: propidium iodide
PS: photosensitizer
RIPK1/RIPK3: Receptor‐interacting protein kinases 1 and 3
ROS: reactive oxygen species

## INTRODUCTION

Gliosarcoma (GS) is a malignant tumor of the central nervous system (CNS), described as rare and with the ability to invade adjacent areas and generate extracranial metastases.^1^, ^2^

Cell death can occur in different ways, depending on the reaction triggered by the therapies applied. Among the various existing therapy options is photodynamic therapy (PDT), a technique based on the interaction of a photosensitizer (PS), light, and molecular oxygen present in the tissues.^3^ PDT involves the irradiation of a PS in the presence of oxygen, generating the formation of reactive oxygen species (ROS), including singlet oxygen. PS in cells can accumulate in different organelles, such as lysosomes, endoplasmic reticulum, and mitochondria, interfering with the cellular response, which is why studying the location of each PS is important. In addition, PDT targets various components of cells, modulating the cytoskeleton, causing DNA damage, and inducing inflammation, so it is necessary to study the effect each PS can have on cell structure after therapy.^4^

Different cellular targets can be reached with PDT, and the target choice depends on the PS affinity for a particular molecule or organelle. Thus, the action of PDT can be observed, for example, in mitochondria, lysosomes, and the plasma membrane. Because it acts on different structures, it can induce various types of cell death.^5^

PDT can be applied in different ways, either systemically or locally, and in parallel with other traditional protocols such as chemotherapy and radiotherapy. Its good therapeutic results have made it a good choice for treating various types of cancer, such as gliomas.^6^

Recent advances in PDT for gliomas show promising approaches; for example, the study involving conjugated polymer nanoparticles proposes metronomic PDT targeting the glioblastoma microenvironment, enhancing therapeutic effectiveness through the controlled release of PS^7^ Additionally, the application of protoporphyrin IX in gliosarcoma cell lines demonstrates significant phototoxicity against tumor cells, improving PDT precision^8^, and another study reveals that combining PDT with pro‐oxidant treatments, like doxorubicin, may amplify therapeutic effects, resulting in more significant tumor growth inhibition and synergistic effects in in vitro environments.^9^

Various types of PS can have a greater affinity for different tumor tissues. For gliomas, chlorin e6 (Ce6) is a much‐studied compound. It is a second‐generation PS with a high affinity for tumor tissues and minor damage to surrounding healthy tissues. The site of accumulation of Ce6 can lead to better efficacy of PDT, and the location can induce different cell death pathways that can trigger necrosis, apoptosis, autophagy, and even activate immunogenic pathways.^10^, ^11^

The PS photoenticine and photodithazine have been shown to sensitize gliosarcoma tumor cells in vitro, decreasing cell viability and leading to morphological differences and ROS production. However, different PS concentrations showed different responses.^12^, ^13^, ^14^, ^15^

Therefore, this study aims to verify the interaction of Ce6, in different concentrations with cellular structures important in the PDT process and related to cell death pathways. It also aims to analyze the morphological changes generated after PDT in treating gliosarcoma.

## MATERIALS AND METHODS

### Cell culture

The 9L/LacZ cell line (BCRJ® CRL‐2200TM) was maintained in DMEM medium (Gibco Sigma—Aldrich) supplemented with 10% fetal bovine serum and 1% penicillin/streptomycin solution (LGC Biotechnology/Gibco™—Thermo Fisher Scientific), conditioned in an incubator at 37°C with 5% CO_2_.

### Photosensitizer

Chlorin e6, Fotoenticine®, was used as a PS (Company Nuevas Tecnologías Científicas—NTC—Llanera—Asturias) from Spain, provided by the Institute of Physics of São Carlos—USP São Carlos—SP—Brazil.

Solubilized and stabilized with 0.5% N‐methyl‐D‐glucosamine. The stock solution concentration was 7.8 mg/mL, from which 1:2 serial dilutions of 200 μg/mL to 6.25 μg/mL were performed with sterile PBS (phosphate‐buffered saline) for the concentrations used in this study, kept in the dark and refrigerated at 4°C.

### Experimental groups

Concentrations of 200, 12.5, and 6.25 μg/mL of chlorine e6 were used and incubated for 1 h without light. The cells were then separated into a control group without irradiation, an LED group, and a group treated with PDT. The LED and PDT groups received a fluence of 10 J/cm^2^ in an LED Biotable (Biopdi – São Carlos, Brazil, 660 nm, 25 mW/cm^2^) for 6 min and 40 s.

### Analysis of mitochondrial membrane potential by JC‐1

After incubation with the PS, 200 μL of JC‐1 (Mitochondrial Membrane Potential Probe, Thermofisher) was added to the control and PDT groups at a concentration of 5 μg/mL and incubated for 15 min to observe qualitative changes in mitochondrial membrane potential (MMP). After incubation, the groups were washed twice with sterile PBS, and 50 μL of Hoescht marker (Thermo Scientific™) for live cells was added at a concentration of 5 μg/mL and incubated again for 30 min. Then, 10 μL of Prolong (Invitrogen™) was added to each well/laminula and taken to the LSM 700 Zeiss confocal microscope to acquire images with monomer excitation at 514 nm and emission at 529 nm and aggregates with excitation at 585 nm and emission at 590 nm.

### Cytoskeleton labeling before and after PDT

Alpha‐tubulin labeling was performed to evaluate qualitative cytoskeletal alterations, the interaction of the PS before PDT, and the changes in the cytoskeleton induced after PDT. The cells were fixed with 4% paraformaldehyde for 10 min and washed with PBS. The cells were then sealed with a 0.25% Triton X‐100 solution in PBS for 15 min. After sealing, the cells were washed with PBS for 3 min, and 0.2% albumin was added and incubated for 45 min. After incubation, the cells were washed with 0.1% albumin in PBS, and 20 μL of the anti‐α‐tubulin antibody (1:500 – Sigma T9026 – 100 μL) diluted in PBS with 0.1% albumin was added and incubated for 15 min.

After the incubation period, the wells were washed with PBS, and the anti‐IgG secondary antibody (1:1000 – Sigma) was added and incubated for 15 min. The cells were washed, and the slides were mounted with a mounting medium containing DAPI (4′,6‐diamidino‐2‐phenylindol), dihydrochloride (Thermofisher – ProLongTM Montante Antifade Diamond with DAPI) for nuclear labeling. The samples were then analyzed under a confocal microscope (LSM 700 Zeiss), with excitation at 488 nm and emission between 500 and 600 nm for DAPI.

### Scanning electron microscopy

Cells were transferred 1 × 10^6^ per well in a 24‐well plate, with a control without PS and the groups with chlorine e6 at the concentrations tested. After 24 h of PDT, the wells were washed with PBS and then dehydrated with ethanol in increasing concentrations: 70% for 15 min, 90% for 15 min, and 100% for 15 min, three times. Finally, a hexadimethyldisilazane (HDMS) drop was added and taken to the incubator at 37°C. The samples were then metalized (Emitech K550X Metallizer). The micrographs were taken using a Zeiss EVO MA 10 Scanning Electron Microscope.

### Flow cytometry analysis

After treatment, cells were trypsinized and resuspended for the experiments. Aliquots of 5 × 10^5^ cells were incubated with the following fluorescent markers: JC‐1 for assessment of MMP, Rhodamine 123 for evaluation of membrane potential, and Annexin V‐FITC and propidium iodide (PI) for detection of apoptosis and necrosis, according to the manufacturer's instructions. After incubation, cells were analyzed on a BD Accuri™ C6 Plus Flow Cytometer with 488 nm and 640 nm laser excitation. Data acquisition was performed using BD software. Data analysis was performed using BD Accuri C6 Plus software, including defining gates to exclude debris and the generation of histograms and dot plots.

## RESULTS

### Analysis of mitochondrial membrane potential by JC‐1

**FIGURE 1:**
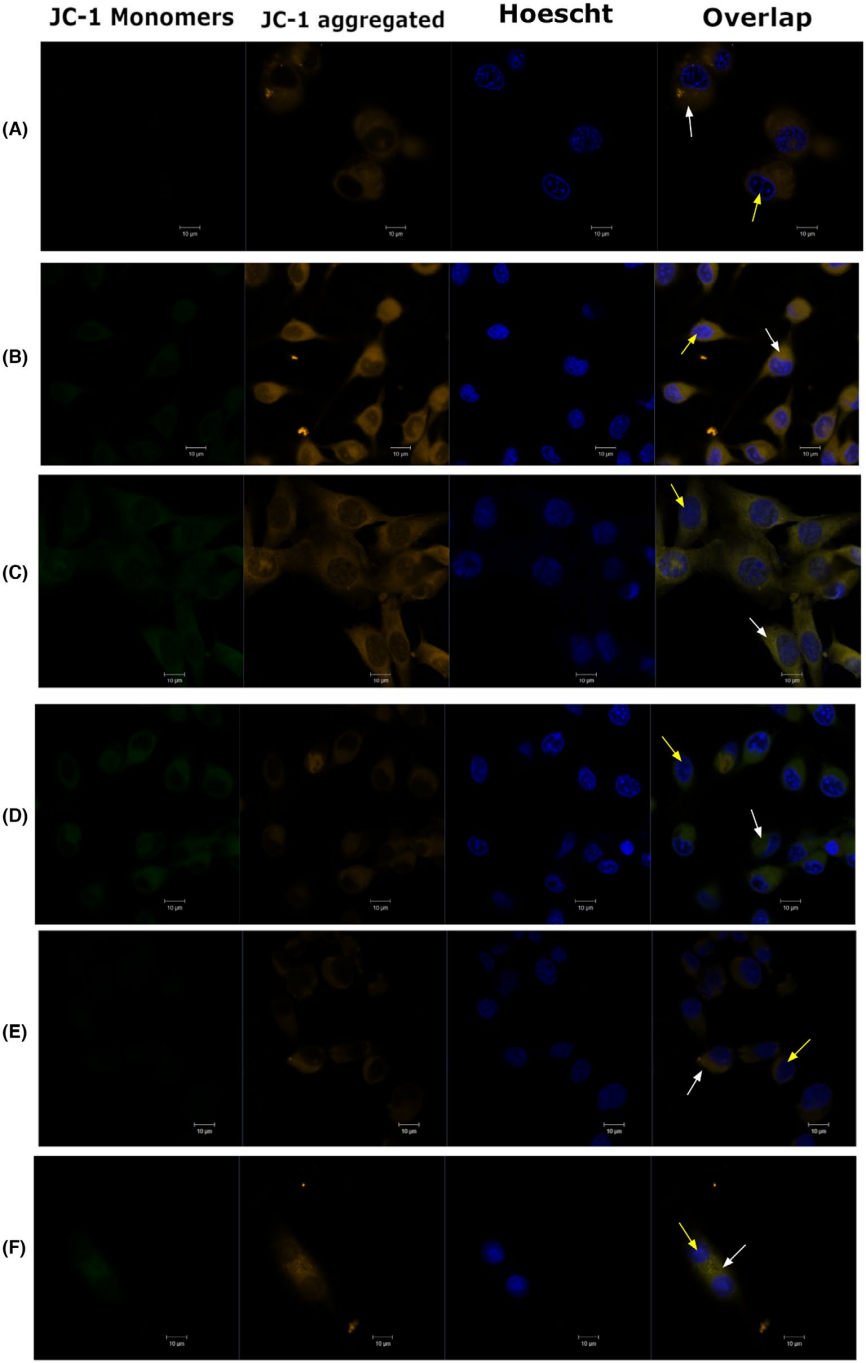
Mitochondrial membrane potential by JC‐1 in confocal microscopy. (A) 200 μg/mL, Dark. (B) 200 μg/mL, PDT. (C) 12.5 μg/mL, Dark. (D) 12.5 μg/mL, PDT. (E) 6.25 μg/mL, Dark. (F) 6.25 μg/mL, PDT. White arrows indicate the cytoplasm, and yellow arrows indicate the nuclei.

In the dark controls, at all concentrations used, the aggregated form of the JC‐1 with orange fluorescence emission predominated, indicating no change in the mitochondria, which remained at a high potential. However, when irradiated with different concentrations of chlorin e6, the treated cells decreased the orange fluorescence signals and increased the green fluorescence signals of the JC‐1 dye, showing a decrease in mitochondrial potential due to the organelles depolarization (Figure 1).

### Cytoskeleton labeling before and after PDT

**FIGURE 2:**
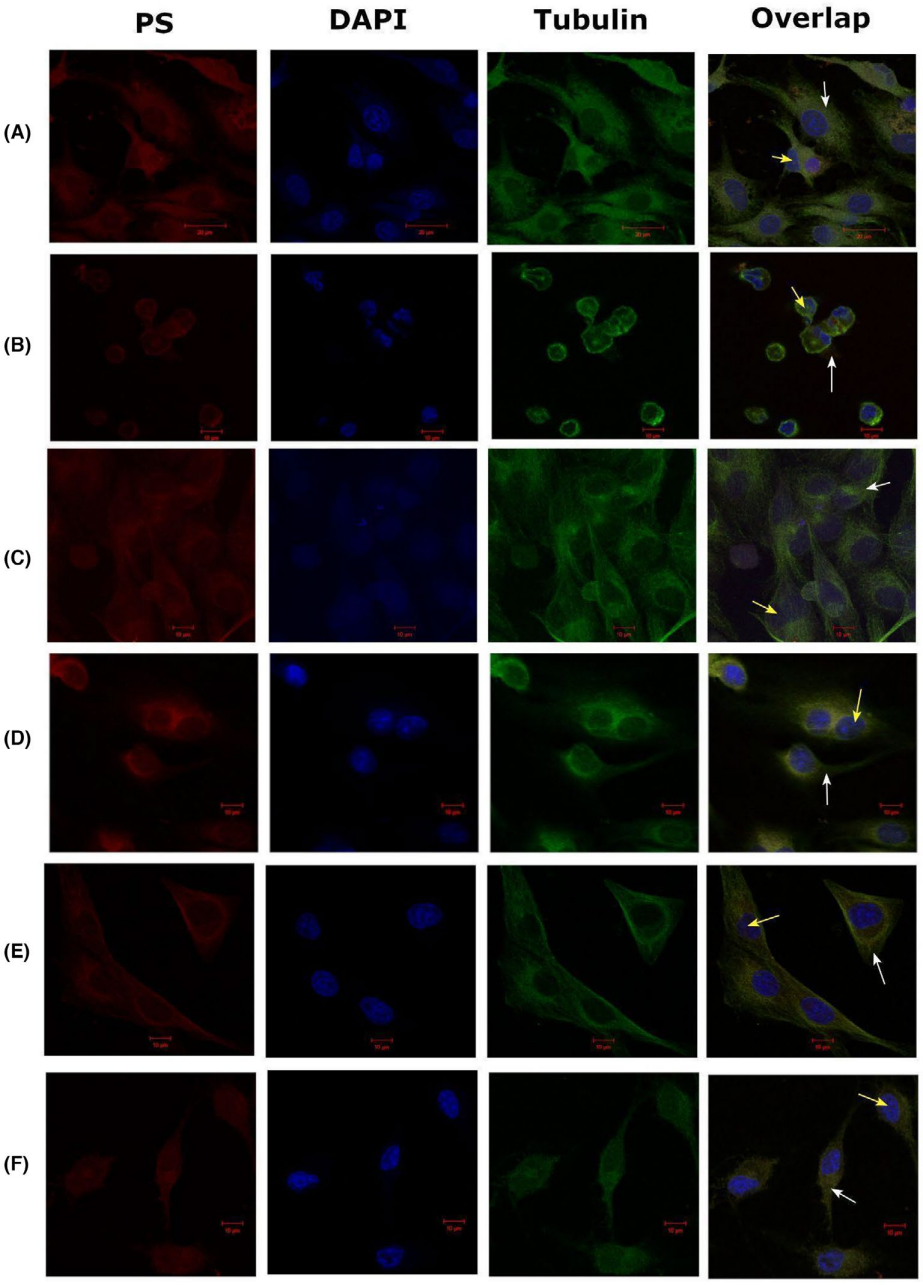
Tubulin labeling before and after PDT in fluorescence confocal microscopy with excitation at 488 nm and emission from 500 nm to 600 nm. (A) 200 μg/mL, Dark. (B) 200 μg/mL, PDT. (C) 12.5 μg/mL, Dark. (D) 12.5 μg/mL, PDT. (E) 6.25 μg/mL, Dark. (F) 6.25 μg/mL, PDT. White arrows indicate cytoplasm and yellow arrows indicate nuclei.

Chlorin interacted with the cytoskeleton at all concentrations, with the PS fluorescence overlapping with the tubulin marking. After PDT, structural alterations were observed in the cytoskeleton, with the most significant changes at the highest concentration, as shown in Figure 2.

### Scanning electron microscopy (SEM)

**FIGURE 3:**
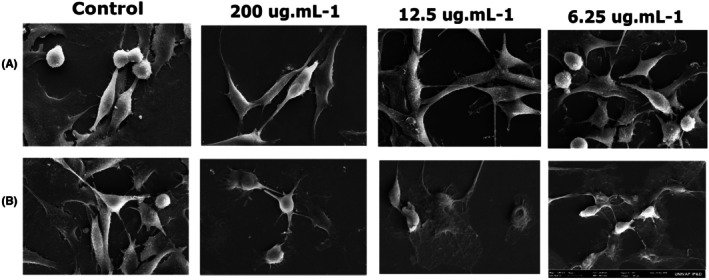
Scanning electron microscopy micrographs of 9L/LacZ cells before and after PDT at 200, 12.5, and 6.25 μg/mL concentrations. Magnification: 1.00KX. (A) Dark. (B) Irradiated.

After 24 h of PDT, the differences between the control and irradiated groups were evident. The control group showed preserved morphology with evident prolongations in the cytoplasm. In the PDT groups, a reduction in cell volume, morphological alterations, and cell fragmentation were observed, as shown in Figure 3.

### Flow cytometry analysis

#### Rhodamine 123

**FIGURE 4:**
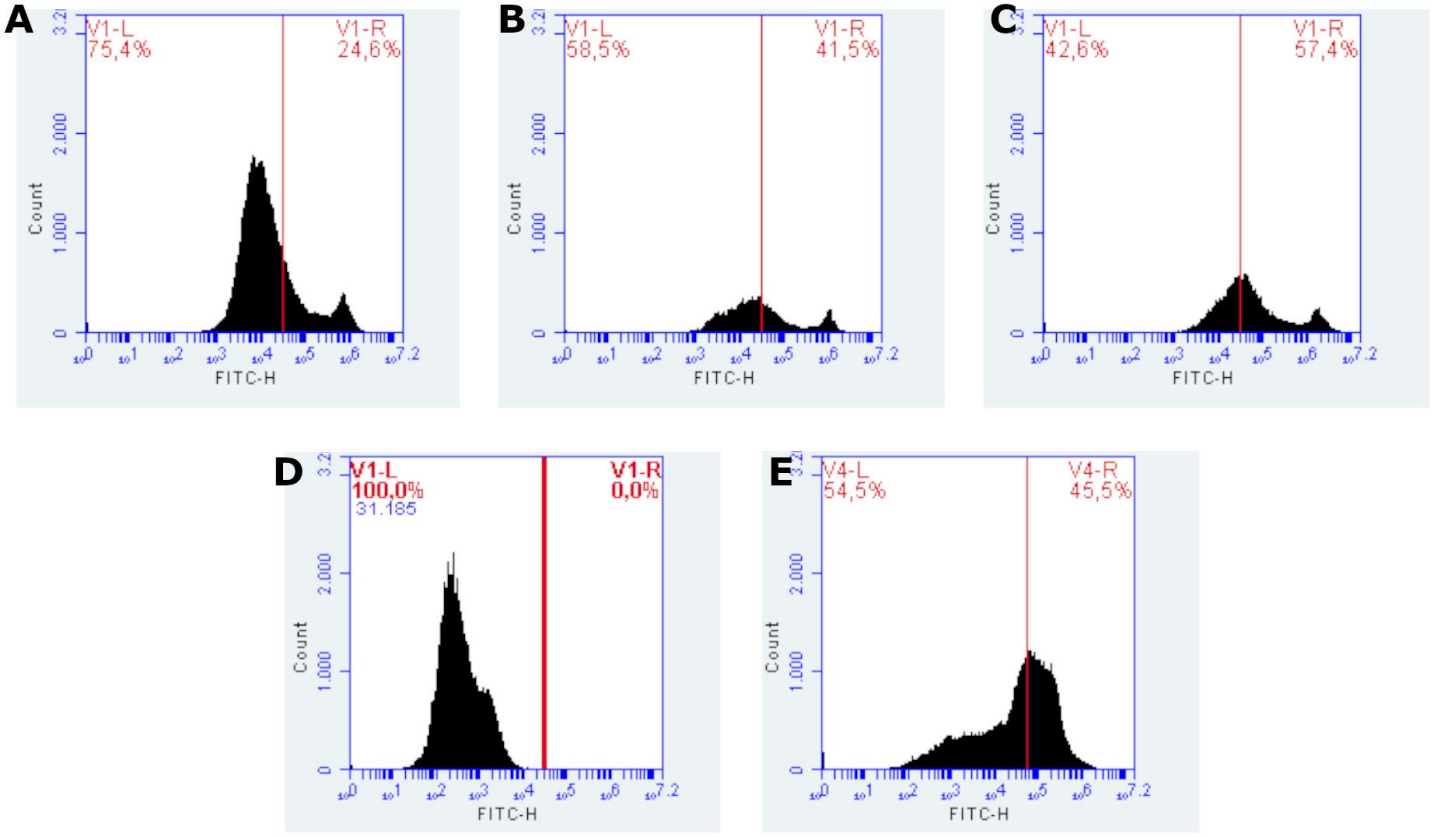
Trimethylated Rhodamine staining of the groups (A) 200 μg/mL, (B) 12.5 μg/mL, (C) 6.25 μg/mL, (D) Depolarized control with H_2_O_2_, and (E) Control.

The analysis of MMP in 9L/lacZ cells treated with PDT with chlorin was performed by flow cytometry, using Rhodamine 123 labeling. This lipophilic fluorescent probe selectively accumulates in mitochondria with preserved membrane potential, allowing the identification of mitochondrial alterations associated with oxidative stress and cell death. The loss of fluorescence is associated with mitochondrial depolarization, an early event in the apoptotic process. The fluorescence histograms revealed different response patterns between the groups evaluated (Figure 4). Cells treated with 200 μg/mL of chlorin presented 24.6% of cells with polarized mitochondria and 75.4% depolarized, indicating that this concentration induced the highest degree of mitochondrial impairment among the treated groups. This suggests that PDT was effective in promoting significant mitochondrial damage in this condition, possibly due to increased generation of ROS. At the concentration of 12.5 μg/mL, 41.5% of cells with polarized mitochondria and 58.5% of depolarized mitochondria were observed, reflecting a significant cytotoxic effect, although less pronounced than at the highest dose. At the lowest concentration tested, 6.25 μg/mL, the proportion of cells with preserved mitochondria was the highest among the treated groups (42.6% depolarized), reducing the impact of PDT in this condition. These data suggest a dose‐dependent relationship between chlorin concentration and the degree of mitochondrial damage induced, with higher concentrations being more effective in depolarizing. The positive control, treated with H_2_O_2_, showed 100% mitochondrial depolarization, confirming the efficacy of the oxidizing agent in inducing membrane potential disturbance. This group was essential to establishing the cutoff limits (gate) between polarized and depolarized populations in the histograms. On the other hand, the negative control (without treatment) showed 54.5% of depolarized cells, a considerably high value for a basal group. This result may indicate the presence of preexisting mitochondrial stress in the experimental conditions, possibly associated with the time of manipulation, exposure to light, or changes in the culture medium. Taken together, the data indicate that a chlorin‐based PDT promotes mitochondrial depolarization in a concentration‐dependent manner, being more effective at doses of 12.5 μg/mL and 200 μg/mL. The integrity of the controls confirms the ability of the methodology to discriminate mitochondrial alterations, although the high depolarization rates in the control group highlight the need to optimize the experimental conditions for greater reproducibility.

#### JC‐1

**FIGURE 5:**
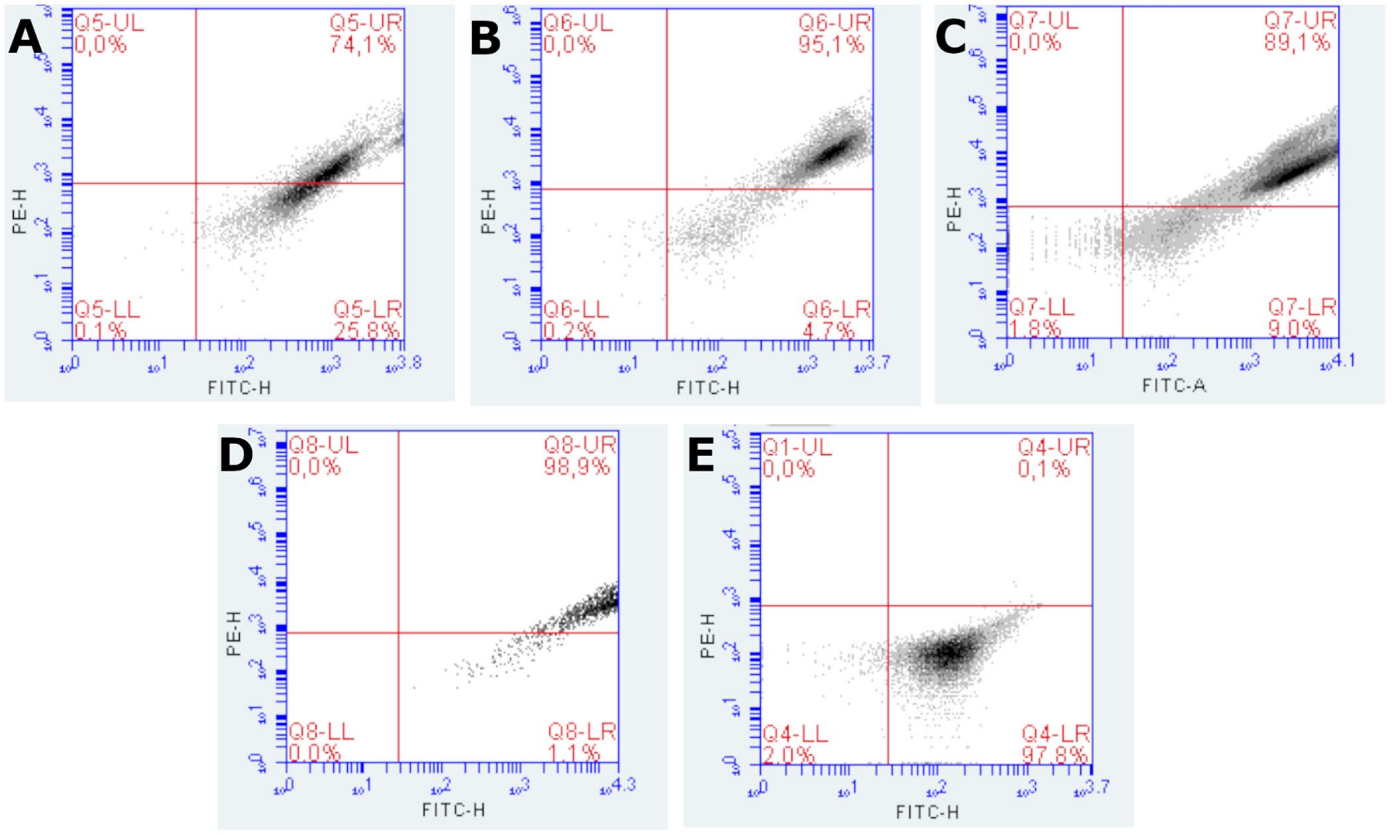
JC‐1 staining of groups (A) 200 μg/mL, (B) 12.5 μg/mL, (C) 6.25 μg/mL, (D) Control, and (E) Depolarized control with H₂O₂.

The assessment of MMP in 9L/lacZ cells subjected to PDT with chlorin was performed by flow cytometry using the JC‐1 dye. This fluorescent marker allows the differentiation between polarized mitochondria, which accumulate the dye in aggregate form (red emission, detected in the PE‐H channel), and depolarized mitochondria, in which the dye remains in monomeric form (green emission, detected in the FITC‐H channel). The scatter plots reveal the distribution of cells in four quadrants, with the upper right quadrant (FITC^+^/PE^+^) representing cells with polarized mitochondria and the lower right quadrant (FITC^+^) representing cells with depolarized mitochondria. This distinction allows quantification of the effects of PDT on mitochondrial integrity. The untreated control group (Graph D) showed 98.9% of cells with polarized mitochondria and 1.1% depolarized, demonstrating preservation of mitochondrial integrity under physiological conditions. In contrast, the group treated with hydrogen peroxide (Graph E), used as a positive control for depolarization, showed 97.8% of cells with depolarized mitochondria and only 0.1% polarized, confirming the effectiveness of the control in inducing mitochondrial potential loss. In the groups subjected to PDT, a response dependent on the PS concentration was observed. The group treated with 200 μg/mL of chlorin (Graph A) showed 74.1% of cells with polarized mitochondria and 25.8% depolarized, evidencing the most pronounced cytotoxic effect among the groups. The group treated with 12.5 μg/mL (Graph B) showed 95.1% of polarized mitochondria and 4.7% depolarized, reflecting an intermediate response. The group treated with 6.25 μg/mL (Graph C) showed 89.1% of polarized cells and 9.0% depolarized, indicating a milder PDT effect at this concentration. The results indicate that PDT with chlorin promotes mitochondrial depolarization in a dose‐dependent manner, with greater efficacy at higher concentrations (200 and 12.5 μg/mL). The lowest concentration tested showed a limited effect, possibly due to not reaching the threshold necessary to trigger efficient photosensitization. The consistency between the data obtained with JC‐1 and those previously presented with trimethylated rhodamine reinforces the central role of mitochondria as the primary target of PDT, highlighting the activation of cell death pathways associated with mitochondrial oxidative stress (Figure 5).

### Annexin V‐FITC and propidium iodide (PI)

**FIGURE 6:**
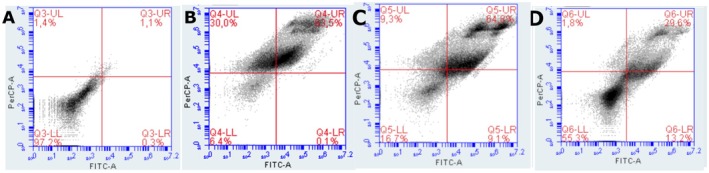
Annexin and propidium iodide staining of the groups control (A), 200 μg/mL (B), 12.5 μg/mL (C), and 6.25 μg/mL (D).

Cell death induced by PDT with chlorin was assessed by flow cytometry using double staining with Annexin V‐FITC and PI, which allows the distinction between viable cells, early apoptotic cells, late apoptotic cells, or necrotic cells. Viable cells are unstained (Annexin−/PI−), early apoptotic cells are positive only for Annexin V (Annexin+/PI−), while late apoptotic or necrotic cells are double‐positive (Annexin+/PI+). In the control sample (without PDT), 97.2% of the cells remained viable, with low levels of early apoptosis (1.4%) and late apoptosis/necrosis (1.1%), establishing a baseline profile of cell viability. After PDT treatment using 200 μg/mL of chlorin, a sharp increase in cell death was observed, with 30.0% of the cells in early apoptosis and 63.5% in late apoptosis/necrosis, leaving only 6.1% of viable cells. This result confirms the high cytotoxic efficacy of PDT at this concentration. On the other hand, treatments with lower doses show less compromise of cell viability. At 12.5 μg/mL of chlorin, 71.3% of cells remained viable, while 26.6% were in late apoptosis/necrosis. A similar profile was observed at the concentration of 6.25 μg/mL, with 67.3% viable cells and 29.6% in late apoptosis/necrosis. In both conditions, the occurrence of early apoptosis was minimal (1.8%). These results indicate that PDT with chlorin induces cell death in a PS concentration‐dependent manner, with late apoptosis being the predominant pattern of cell death observed under the tested conditions. A concentration of 200 μg/mL stands out for promoting the highest rate of cell compromise, while lower concentrations preserve a specific portion of viable cells, with a more moderate cytotoxic effect (Figure 6).

## DISCUSSION

PDT with chlorin e6 (Ce6) induced cell damage in the 9L/LacZ gliosarcoma cell line across all tested concentrations, with varying effects among groups. Similar studies with photodithazine and photoenticine demonstrated PDT potential to damage gliosarcoma cells, with Ce6 showing no dark cytotoxicity but higher cell death rates at elevated concentrations.^12^, ^13^, ^14^

The efficacy of PDT is closely linked to the induction of various regulated cell death pathways, which are activated in response to oxidative stress and depend on the type and subcellular localization of the PS.^16^, ^17^ Among the best‐characterized mechanisms triggered by PDT, apoptosis is one of the main forms. This programmed cell death can be initiated by the extrinsic pathway, mediated by death receptors such as Fas and TNFR, or by the intrinsic pathway, involving mitochondrial dysfunction, cytochrome c release, and caspase‐9 activation, leading to the activation of effector caspases like caspase‐3.^18^, ^19^ An increase in the expression of proapoptotic proteins (such as Bax) and a reduction of antiapoptotic proteins (such as Bcl‐2) have been observed in tumor models treated with PDT, confirming this apoptotic profile.^19^

In addition to these pathways, PDT can also trigger necroptosis, a programmed necrosis process regulated by RIPK1/RIPK3/MLKL, which leads to plasma membrane rupture and inflammatory signaling (Pashootan et al., 2024). Pyroptosis and parthanatos have also been described as possible outcomes induced by PDT through mechanisms involving PARP‐1 hyperactivation and inflammasome activation.^16^ Autophagy is frequently activated as an initial cellular response to the oxidative stress caused by PDT, functioning as a cytoprotective mechanism. However, when exacerbated or dysregulated, autophagy may contribute to cell death.^20^ The accumulation of autophagosomes and the failure of lysosomal degradation are key steps in this transition from protective to lethal autophagy.

Ferroptosis, a distinct form of iron‐dependent cell death, is characterized by the accumulation of lipid peroxides and failure of the enzyme glutathione peroxidase 4 (GPX4), leading to destabilization of cellular membranes.^21^ PDT can induce ferroptosis through depletion of glutathione (GSH), inhibition of the system Xc^−^ antiporter, and increased ROS generation, especially in tumor cells highly sensitive to oxidative stress.^19^, ^20^

Another relevant feature of PDT is its ability to induce immunogenic cell death (ICD). This form activates antitumor immune responses by promoting the exposure and release of damage‐associated molecular patterns (DAMPs), such as calreticulin, ATP, and HMGB1, which stimulate dendritic cell maturation and tumor‐specific T lymphocyte responses.^16^, ^20^ Therefore, PDT demonstrates a multifaceted profile of regulated cell death induction, including apoptosis, ferroptosis, necroptosis, autophagy, and ICD. The predominance of each pathway depends on the cell type, the PS used, and the intensity of oxidative stress.^17^, ^20^

PDT emerges as a promising strategy in glioma treatment, combining direct cell death with immune response modulation. PDT induces tumor cell death by generating ROS upon PS activation by light, leading to apoptosis, a programmed and less inflammatory cell death process, and necrosis, which can trigger an inflammatory response. Additionally, PDT promotes the release of DAMPs, which stimulate antigen presentation by antigen‐presenting cells (APCs) and the activation of CD8+ T cells, strengthening immunological surveillance against the tumor. Thus, PDT demonstrates therapeutic potential by attacking glioblastoma through multiple pathways, inducing direct cell death and, simultaneously, orchestrating an antitumor immune response, although translating these findings into clinical practice still requires further investigation.^22^, ^23^

Mitochondria are a key target, as changes in membrane potential and electrochemical gradients can trigger apoptotic cascades.^24^, ^25^ While mitochondria are primary PDT targets, plasma membranes and lysosomes also play roles. Mitochondrial localization favors apoptosis, plasma membrane localization induces necrosis, and lysosomal localization promotes autophagy.^2^, ^10^

The integrity of mitochondria was evaluated by two fluorescent probes, JC‐1 and Rhodamine 123, which revealed consistent but distinct results. The JC‐1 marker was used to identify variations in mitochondrial transmembrane potential. The loss of MMP is considered a substantial parameter of cell function. Thus, measuring changes in MMP is very important to follow the apoptotic process. It is known that in cell microenvironments with standard membrane potential, JC‐1 accumulates in energized and negatively charged mitochondria, forming red fluorescent aggregates. In contrast, in cells undergoing cell death, JC‐1 penetrates the mitochondria to a lesser extent due to increased membrane permeability and loss of electrochemical gradient, maintaining the form of monomers with green fluorescence emission.^24^, ^26^

JC‐1 staining, analyzed by both confocal microscopy and flow cytometry, showed a dose‐dependent mitochondrial depolarization following PDT with Ce6. At 200 μg/mL, a significant shift from red aggregates to green monomers indicated a profound loss of mitochondrial potential, while lower concentrations showed a milder effect. Flow cytometry reinforced these findings, with the highest concentration showing the greatest percentage of depolarized cells. Notably, the 12.5 μg/mL and 6.25 μg/mL groups preserved higher proportions of polarized mitochondria, suggesting partial resistance or delayed damage progression. These findings suggest that the highest tested concentration had a more detrimental effect on the cells and aligned with Almeida et al. 2020, who demonstrated chlorin e6 accumulation in a gliosarcoma cell line mitochondria using Rhodamine 123 staining and MTT assay.

Rhodamine 123 analysis confirmed the general pattern of mitochondrial depolarization across PDT‐treated groups but revealed a discrepancy in the control group. While JC‐1 detected only 1.1% of depolarized cells in untreated controls, Rhodamine indicated 54.5% depolarization. This divergence may stem from the different mechanisms of dye accumulation and fluorescence emission: JC‐1 forms potential‐dependent aggregates that are more stable, whereas Rhodamine 123 is more susceptible to transient fluctuations and experimental variations. These findings underscore the necessity of combining complementary assays to reliably assess mitochondrial function.

Despite the overall consistency between JC‐1 and Rhodamine 123, the significant discrepancy in the control group highlights a methodological consideration. JC‐1 showed 98.9% of cells with polarized mitochondria in the untreated control group, while Rhodamine 123 suggested over half were depolarized. This may result from the more transient and sensitive nature of Rhodamine 123 staining, which is more prone to experimental variation such as handling stress, incubation time, or exposure to light. In contrast, JC‐1 provides a more stable signal due to aggregate formation. These differences emphasize the importance of using multiple and complementary approaches to assess mitochondrial integrity and reinforce the need for rigorous standardization of flow cytometry protocols.

The impact on mitochondrial activity may influence the type of cell death observed. Therefore, annexin and PI were used to investigate each concentration's predominant cell death pathway. Flow cytometry analysis with Annexin V/PI demonstrated that PDT with Ce6 induced cell death in a concentration‐dependent manner, with a predominance of apoptosis at intermediate concentrations and late necrosis at higher doses. At 200 μg/mL, the highest rate of cell death was observed, with 63.5% of cells in late apoptosis/necrosis and 30.0% in early apoptosis, indicating severe mitochondrial collapse. At lower concentrations, such as 12.5 and 6.25 μg/mL, a higher percentage of viable cells was preserved, although mitochondrial impairment and apoptotic initiation were still evident. These findings support that mitochondrial damage is an early and decisive event in Ce6‐mediated PDT, influencing the pattern of cell death observed.

The necrotic pathway shows that the cells have been exposed to severe damage since this type of cell death is considered a form of rapid response and is not regulated by specific signaling pathways. Apoptosis, on the other hand, is a regulated form of death triggered by intracellular or extracellular disturbances involving the activation of specific caspases and causing cell shrinkage, chromatin condensation, and the formation of apoptotic bodies. Late apoptosis is when cells undergo significant morphological and biochemical changes and can produce pro‐inflammatory cytokines. In addition, the plasma membrane becomes more permeable, leading to the exposure of molecules and the release of intracellular content.^27^, ^28^

This variation in the cell death pathway was also observed in the application of PDT with photodithazine and photoenticine on the same gliosarcoma cell line. PDT with photoenticine showed a predominance of necrosis followed by late apoptosis, but this pattern changed with photodithazine. In this case, late apoptosis was observed after PDT as the main death pathway, followed by the necrotic pathway, demonstrating an important difference in the behavior of different PSs regarding gliosarcoma.^8^

In addition to the concentration of PS, the type of damage caused to the cytoskeleton components also influences the mechanism of death induced by PDT. The cytoskeleton is a network of polymerized proteins comprising microfilaments, microtubules, and intermediate filaments, which play an important role in maintaining cell morphology and cell division. In a normal microenvironment, the components of the cytoskeleton are highly integrated and have coordinated functions; however, when the cytoskeleton is destroyed, its functioning is impaired.^4^, ^29^

The interaction of the PS with the cytoskeleton before and after PDT, as well as the analysis of the effects that the therapy can cause on the structural organization of the cell, was observed through fluorescent labeling of tubulin. This protein polymerizes to form microtubules and is analyzed by scanning electron microscopy.

Chlorin e6 interacted with tubulin at all concentrations, but it was clear that after PDT, changes occurred in the cytoskeleton of the treated cells, and in the 200 μg/mL group, these changes were more intense. Scanning electron microscopy also showed morphological changes in the treated groups compared with the control groups. In the cells treated at the highest concentration, loss of the spindle shape was observed, and the cells became rounded. Tubulin is still observed in the fluorescent staining, but structurally, its organization is affected.

This structural loss of the cytoskeleton, characterized by shrinkage in size compared to cells in the control group, is also observed after PDT with photodithazine in 9L/LacZ and glioblastoma lineages, demonstrating that PDT can influence both types of gliomas.^12^, ^30^, ^31^

PDT at 200 μg/mL triggered more significant cell damage and activation of the necrotic pathway, indicating immediate cell damage. Decreasing the concentration reduces this damage and triggers controlled death. Therefore, PDT in association with Ce6 at lower concentrations is possibly a better option for clinical use because the apoptosis death pathway is a regulated pathway with more moderate damage.

The PS selection is one of the main determinants of clinical efficacy in PDT. PSs vary in their ability to generate ROS, tumor selectivity, light absorption characteristics, and the specific cell death pathways they activate.^20^

The Fotoenticine, a chlorin‐based second‐generation PS, presents several advantages compared to first‐generation agents, such as Photofrin®. Fotoenticine absorbs light at 660 nm, allowing deeper tissue penetration and greater tumor selectivity.^16^, ^20^ Its preferential subcellular localization in mitochondria and lysosomes facilitates the activation of mitochondrial apoptosis and ferroptosis.^19^ Studies have shown that Fotoenticine leads to caspase‐3 activation, increased Bax expression, decreased Bcl‐2 levels, and suppression of GPX4 activity—key molecular events in the induction of apoptosis and ferroptosis.^19^, ^21^ Unlike traditional PSs like Photofrin®, which often cause necrosis due to excessive extracellular ROS, Fotoenticine promotes regulated cell death, which is desirable in oncology for minimizing inflammation and off‐target tissue damage.^16^, ^18^ Fotoenticine has also demonstrated potential to induce ICD by exposing calreticulin and promoting the release of ATP and HMGB1, thereby stimulating the immune system against the tumor.^32^

Other PSs, such as HiPorfin, composed of hematoporphyrin derivatives, have also shown efficacy in inducing apoptosis and ferroptosis, particularly by modulating the P53/SLC7A11/GPX4 axis in cholangiocarcinoma cells.^19^ However, Fotoenticine distinguishes itself by exhibiting lower systemic toxicity and faster clearance, making it a safer and more effective option.^16^ In summary, Fotoenticine is a promising PS in PDT due to its capacity to induce multiple forms of regulated cell death, improve immune activation, and reduce systemic side effects. Its superiority over earlier generation PSs places it at the forefront of modern strategies in cancer phototherapy.^16^, ^20^

## CONCLUSION

The results obtained in the study show that chlorin e6 can be a potential PS for treating gliosarcoma using PDT. After PDT, it demonstrates its ability to promote cellular and structural alterations, inducing cell death by necrosis and apoptosis pathways and, consequently, the destruction of the gliosarcoma in all concentrations. The analyzed concentrations of chlorin e6 showed that, regardless of the dose used, gliosarcoma cells are sensitive to this PS. Still, lower doses demonstrate the ability to permeabilize the plasma membrane, structural changes, and increase the percentage of the late apoptosis pathway, which is recommended for clinical use.

## AUTHOR CONTRIBUTIONS

**Marina Gabriela Teixeira:** data curation, writing – original draft. **Rainara Moreno Sanches de Almeida:** funding acquisition, formal analysis. **Juliana Guerra Pinto:** conceptualization, formal analysis, writing – review and editing. **Isabelle Ferreira:** writing – review and editing. **Juliana Ferreira‐Strixino:** conceptualization, resources, writing – review and editing, supervision, project administration, funding acquisition.

## FUNDING INFORMATION

This research was funded by the São Paulo State Research Support Foundation (FAPESP), grant numbers 2016/12211‐4 and 2018/15302‐6.

## Data Availability

Research data are not shared.

## References

[1] Saadeh F , El Iskandarani S , Najjar M , Assi HI . Prognosis and management of gliosarcoma patients: a review of literature. Clin Neurol Neurosurg. 2019;182:98‐103. doi:10.1016/j.clineuro.2019.05.008 31112812

[2] Alexiou G , Hsia T , Small JL , et al. Systematic review of photodynamic therapy in gliomas. Cancer. 2023;15:3918. doi:10.3390/cancers PMC10417382 37568734

[3] Miretti M , González Graglia MA , Suárez AI , Prucca CG . Photodynamic therapy for glioblastoma: a light at the end of the tunnel. J Photochem Photobiol. 2023;13:100161. doi:10.1016/j.jpap.2023.100161

[4] Dubey T , Chinnathambi S . Photodynamic sensitizers modulate cytoskeleton structural dynamics in neuronal cells. Cytoskeleton. 2021;78:232‐248. doi:10.1002/cm.21655 33641243

[5] Castano AP , Demidova TN , Hamblin MR . Mechanisms in photodynamic therapy: part one – photosensitizers, photochemistry and cellular localization. Photodiagn Photodyn Ther. 2004;1:279‐293. doi:10.1016/S1572-1000(05)00007-4 PMC4108220 25048432

[6] Kwiatkowski S , Knap B , Przystupski D , et al. Photodynamic therapy – mechanisms, photosensitizers and combinations. Biomed Pharmacother. 2018;106:1098‐1107. doi:10.1016/j.biopha.2018.07.049 30119176

[7] Caverzán MD , Oliveda PM , Beaugé L , Palacios RE , Chesta CA , Ibarra LE . Metronomic photodynamic therapy with conjugated polymer nanoparticles in glioblastoma tumor microenvironment. Cells. 2023;12:1541. doi:10.3390/cells12111541 37296661 PMC10252555

[8] Fontana LC , Pinto JG , Magalhães JA , et al. Comparison of the photodynamic effect of two chlorins, photodithazine and fotoenticine, in gliosarcoma cells. Photochem. 2022;2:165‐180. doi:10.3390/photochem2010013

[9] Cesca BA , Caverzan MD , Lamberti MJ , Ibarra LE . Enhancing therapeutic approaches in glioblastoma with pro‐oxidant treatments and synergistic combinations: in vitro experience of doxorubicin and photodynamic therapy. Int J Mol Sci. 2024;25:7525. doi:10.3390/ijms25147525 39062770 PMC11277534

[10] Yang K , Niu T , Luo M , Tang L , Kang L . Enhanced cytotoxicity and apoptosis through inhibiting autophagy in metastatic potential colon cancer SW620 cells treated with chlorin e6 photodynamic therapy. Photodiagn Photodyn Ther. 2018;24:332‐341. doi:10.1016/j.pdpdt.2018.10.012 30355513

[11] Zhang ZJ , Wang KP , Mo JG , Xiong L , Wen Y . Photodynamic therapy regulates fate of cancer stem cells through reactive oxygen species. World J Stem Cells. 2020;12:562‐584. doi:10.4252/wjsc.v12.i7.562 32843914 PMC7415247

[12] dos Santos Vitorio G , de Almeida RMS , Pinto JG , Fontana LC , Ferreira‐Strixino J . Analysis of the effects of photodynamic therapy with Photodithazine on the treatment of 9l/lacZ cells, in vitro study. Photodiagn Photodyn Ther. 2021;34:102233. doi:10.1016/j.pdpdt.2021.102233 33639321

[13] Fontana LC , Pinto JG , Pereira AHC , Soares CP , Raniero LJ , Ferreira‐Strixino J . Photodithazine photodynamic effect on viability of 9L/lacZ gliosarcoma cell line. Lasers Med Sci. 2017;32:1245‐1252. doi:10.1007/s10103-017-2227-5 28503718

[14] de Almeida RMS , Fontana LC , dos Santos Vitorio G , et al. Analysis of the effect of photodynamic therapy with Fotoenticine on gliosarcoma cells. Photodiagn Photodyn Ther. 2020;30:101685. doi:10.1016/j.pdpdt.2020.101685 32050104

[15] Fontana LC , Pinto JG , Vitorio GDS , et al. Photodynamic effect of protoporphyrin IX in gliosarcoma 9l/lacZ cell line. Photodiagn Photodyn Ther. 2022;37:102669. doi:10.1016/j.pdpdt.2021.102669 34863947

[16] Mishchenko T , Balalaeva I , Gorokhova A , Vedunova M , Krysko DV . Which cell death modality wins the contest for photodynamic therapy of cancer? Cell Death Dis. 2022;13:455. doi:10.1038/s41419-022-04851-4 35562364 PMC9106666

[17] Broekgaarden M , Weijer R , van Gulik TM , Hamblin MR , Heger M . Tumor cell survival pathways activated by photodynamic therapy: a molecular basis for pharmacological inhibition strategies. Cancer Metastasis Rev. 2015;34:643‐690. doi:10.1007/s10555-015-9588-7 26516076 PMC4661210

[18] Elmore S . Apoptosis: a review of programmed cell death. Toxicol Pathol. 2007;35:495‐516. 17562483 10.1080/01926230701320337 PMC2117903

[19] Yan X , Li Z , Chen H , Yang F , Tian Q , Zhang Y . Photodynamic therapy inhibits cancer progression and induces ferroptosis and apoptosis by targeting P53/GPX4/SLC7A11 signaling pathways in cholangiocarcinoma. Photodiagn Photodyn Ther. 2024;47:104104. doi:10.1016/j.pdpdt.2024.104104 38679154

[20] Pashootan P , Saadati F , Fahimi H , et al. Metal‐based nanoparticles in cancer therapy: exploring photodynamic therapy and its interplay with regulated cell death pathways. Int J Pharm. 2024;649:123622. doi:10.1016/j.ijpharm.2023.123622 37989403

[21] Costa I , Barbosa DJ , Benfeito S , et al. Molecular mechanisms of ferroptosis and their involvement in brain diseases. Pharmacol Ther. 2023;244:108373. doi:10.1016/j.pharmthera.2023.108373 36894028

[22] Bartusik‐Aebisher D , Żołyniak A , Barnaś E , et al. The use of photodynamic therapy in the treatment of brain tumors—a review of the literature. Molecules. 2022;27:6847. doi:10.3390/molecules27206847 36296440 PMC9607067

[23] Aebisher D , Przygórzewska A , Myśliwiec A , et al. Current photodynamic therapy for glioma treatment: an update. Biomedicine. 2024;12:375. doi:10.3390/biomedicines12020375 PMC10886821 38397977

[24] Sivandzade F , Bhalerao A , Cucullo L . Analysis of the mitochondrial membrane potential using the cationic JC‐1 dye as a sensitive fluorescent probe. Bio Protoc. 2019;9:e3128. doi:10.21769/BioProtoc.3128. PMC6343665 30687773

[25] Yue J , Shen Y , Liang L , et al. Revealing mitochondrial microenvironmental evolution triggered by photodynamic therapy. Anal Chem. 2020;92:6081‐6087. doi:10.1021/acs.analchem.0c00497 32208680

[26] Zhang CH , Wang S , Zhang P , et al. Cellular and mitochondrial dual‐targeted nanoprobe with near‐infrared emission for activatable tumor imaging and photodynamic therapy. Sens Actuators B Chem. 2021;346:130451. doi:10.1016/j.snb.2021.130451

[27] Poon IKH , Hulett MD , Parish CR . Molecular mechanisms of late apoptotic/necrotic cell clearance. Cell Death Differ. 2010;17:381‐397. doi:10.1038/cdd.2009.195 20019744

[28] Donohoe C , Senge MO , Arnaut LG , Gomes‐da‐Silva LC . Cell death in photodynamic therapy: from oxidative stress to anti‐tumor immunity. Biochim Biophys Acta Rev Cancer. 2019;1872:188308. doi:10.1016/j.bbcan.2019.07.003 31401103

[29] Ma H , Yang K , Li H , Luo M , Wufuer R , Kang L . Photodynamic effect of chlorin e6 on cytoskeleton protein of human colon cancer SW480 cells. Photodiagn Photodyn Ther. 2021;33:102201. doi:10.1016/j.pdpdt.2021.102201 33529743

[30] Karmakar S , Banik NL , Patel SJ , Ray SK . 5‐Aminolevulinic acid‐based photodynamic therapy suppressed survival factors and activated proteases for apoptosis in human glioblastoma U87MG cells. Neurosci Lett. 2007;415:242‐247. doi:10.1016/j.neulet.2007.01.071 17335970 PMC2533742

[31] Castilho‐Fernandes A , Lopes TG , Primo FL , Pinto MR , Tedesco AC . Photodynamic process induced by chloro‐aluminum phthalocyanine nanoemulsion in glioblastoma. Photodiagn Photodyn Ther. 2017;19:221‐228. doi:10.1016/j.pdpdt.2017.05.003 28599959

[32] Garg AD , Dudek‐Peric AM , Romano E , Agostinis P . Immunogenic cell death. Int J Dev Biol. 2015;59:131‐140. doi:10.1387/ijdb.150061pa 26374534

